# Effects of aging and valence on emotional response inhibition: conclusions from a novel stop-signal task

**DOI:** 10.3389/fpsyg.2025.1568492

**Published:** 2025-04-09

**Authors:** Jill D. Waring, Stephanie N. Hartling

**Affiliations:** Department of Psychology, Saint Louis University, St. Louis, MO, United States

**Keywords:** response inhibition, emotion, aging, stop-signal task, inhibitory control, executive functioning

## Abstract

Emotional and cognitive processes interact in myriad ways during daily life, and the relation between emotion and cognition changes across the lifespan. Aging is associated with decreasing cognitive control and inhibition alongside improvements in emotional control and regulation. However, little is known about how aging impacts response inhibition within emotionally relevant contexts. The current study examined how aging impacts emotional response inhibition by comparing older and younger adults’ ability to stop responses to emotional images. Participants completed a novel stop-signal task where pleasant and unpleasant scene images appeared on a minority of trials, while participants developed a pre-potent ‘go’ response during trials presenting neutral shapes. Notably, in each task block only one of the two types of emotional scene images served as a task-relevant stop cue, e.g., unpleasant images as stop-signals. Accordingly, in a given task block participants should continue to respond at the onset of the other type of emotional image (i.e., pleasant scenes as ‘go-images’). Overall, older adults exhibited less efficient stopping than younger adults. However, stopping did not differ between pleasant and unpleasant images in either age group. Thus, while response inhibition is less efficient in older adults, it does not differ by emotion across adulthood. The innovative design also permitted exploratory analyses of responses to images that were not the current stop-signal, i.e., responses correctly executed for ‘go-image’ trials. In contrast with response inhibition on stop trials, emotion and aging significantly interacted during response execution, with older adults performing less accurately than younger adults on unpleasant go-image trials. Taken together, aging interacts with emotion only for response execution but not response inhibition for emotional scenes. This study offers new insights into the effects of aging on response inhibition in emotionally complex contexts and increases the ecological validity of response inhibition research. It also highlights the distinct effects of aging and emotion on response execution versus inhibition for task-relevant emotional information.

## 1 Introduction

Like many cities and towns, St. Louis has many busy streets with impatient drivers who often disregard traffic signals. To safely traverse the streets pedestrians must vigilantly detect and respond to traffic conditions, often including stopping abruptly to avoid collision with cars. Such actions demonstrate reactive inhibition, that is, stopping ongoing movements to dynamically respond to the environment and maintain safety while pursuing current goals (Aron, 2011). In laboratory settings, the most precise and widely used paradigm to measure such response inhibition is the stop-signal task, which requires stopping or canceling responses already in progress (Logan, 1994; Verbruggen et al., 2019). Thus, the cognitive processes engaged in the stop-signal task resemble real-world decisions, such as deciding whether to stop walking when an approaching car speeds through a red traffic signal or to continue crossing the street if the car is slowing and it seems safe to proceed walking.

### 1.1 Effects of emotion on reactive response inhibition

Although exhaustive investigations into response inhibition have yielded valuable information about nuanced factors influencing inhibitory control abilities, they have done so largely in contexts devoid of affective relevance (Verbruggen and Logan, 2009; Wessel, 2025). This leaves many open questions about how humans engage in response inhibition upon encountering emotional information. In everyday life we rarely inhibit or complete a behavior in response to explicit unambiguous cues lacking any affective salience. Yet, this is the scenario modeled in most laboratory tasks assessing inhibitory control (Hannah and Aron, 2021). Notably, even commonly cited examples of response inhibition in the real world, such as the traffic scenario described earlier (Matzke et al., 2018; Verbruggen and Logan, 2009; Wessel, 2018, 2025) depict an affectively relevant context, i.e., evaluating the relative safety or danger of the options to stop for oncoming traffic or continue walking. Despite the abundance of empirical studies of response inhibition, relatively very few have considered the direct influence of emotional information or affective salience on stopping performance.

Of the limited investigations into how emotion impacts response inhibition, many have focused on the carry-over or priming effect of exposure to emotional (positive and/or negative) images interspersed between trials in a standard stop-signal task. In the standard stop-signal task participants initiate a motor response to visual stimuli on the screen, and in about 75% of trials the correct action is to complete the response (“go trials”) while the remaining minority of trials require stopping the movement underway at the onset of a cue to stop (“stop trials”) to cancel the response. Results indicate that viewing emotional images (versus neutral images) immediately before go trials featuring arrows, shapes, or letters typically impairs stopping performance for the sporadic onset of an auditory tone or color change stop-cue (Kalanthroff et al., 2013; Krypotos et al., 2011; Patterson et al., 2016; Verbruggen and De Houwer, 2007; Wang et al., 2024). But see Littman and Takács (2017) for evidence that inhibition improved following negative images. Studies with this design generally indicate that emotion processing effectively interrupts or usurps mental resources, leaving fewer resources available for inhibitory control in the subsequent stop-trial. Another approach employs emotional facial expressions, images, or words in go trials in conjunction with a standard auditory tone or color cue as the stop-signal (i.e., ‘implicit’ emotional stop-signal task; Battaglia et al., 2021; Ding et al., 2020). This design has yielded highly inconsistent effects of emotion ranging from impairing, enhancing, or having no effect on response inhibition (Camfield et al., 2018; Herbert and Sütterlin, 2011; Nayak et al., 2019; Pawliczek et al., 2013; Rebetez et al., 2015; Sagaspe et al., 2011). These mixed results suggest that the implicit emotional stop-signal task may be especially sensitive to specific task design attributes, such as the complexity of the go-task demands (Ding et al., 2020).

Recent innovations of the stop-signal task have increasingly incorporated emotional content (e.g., facial expressions, images, body poses, sounds) as the stop-signals (i.e., ‘explicit’ emotional stop-signal task; Battaglia et al., 2021; Ding et al., 2020). However, in nearly all cases the emotional content of the stop-signal was irrelevant to correctly following task instructions. Meaning, instructions have not required an overt focus on emotion to make the correct stopping decision. For example, participants are typically instructed to stop their responses whenever a face appears after a go-signal, irrespective of the specific facial expression displayed. Results from these designs indicate that incidental presence of emotion within stop trials—emotion is not task-relevant for stopping—typically improves response inhibition (Battaglia et al., 2022b, 2022a; Gupta and Singh, 2021; Pandey and Gupta, 2022b, 2022a; Pessoa et al., 2012; Senderecka, 2016, 2018).^1^

Our prior study (Williams et al., 2020) remains the only investigation to date that required direct consideration of the emotional content of the stop-signal to determine whether to stop an ongoing response, i.e., emotion was task-relevant. Across three studies we identified that emotion (positive or negative, vs. neutral) significantly influences inhibitory control only when it is necessary to consider emotion to implement stop-trial instructions correctly. We observed better response inhibition for happy-face stop-signals compared to fearful-face stop-signals, but only when participants needed to focus directly on the emotional attributes of the facial expressions to make their stopping decisions. This effect was absent after instructions to stop for any face or stop based on the gender (male or female) of the face. As the literature shows, with the exception of Williams et al. (2020), discernment and interpretation of emotional content in stop-signal tasks has not been necessary to make the correct stopping decision, i.e., emotion has generally not been task-relevant for implementing inhibition. Consequently, although much of everyday inhibitory control is implemented in emotionally salient contexts, much is still unknown about response inhibition performance when the choice to stop (or not) depends upon the specific emotional content encountered.

Additional context for appreciating the role of task relevance in an emotional stop-signal task can be drawn from emotional variants of the go/no-go task, although the cognitive demands differ somewhat across varieties of emotional response inhibition tasks (Meyer and Bucci, 2016; Raud et al., 2020; Verbruggen and Logan, 2008). Investigations employing go/no-go tasks have indicated that when focus on emotion is task-relevant, positive facial expressions and body postures (but not negative ones) impair inhibitory control in younger adults (Calbi et al., 2022; Mancini et al., 2022). Notably, the distinct effects of emotion were not observed when task instructions drew attention to non-emotional aspects of the stimuli (e.g., gender). Thus, while the direction of the effect of emotion differs between the stop-signal task and go/no-go task literature, evidence from both tasks indicates a significant role of task-relevance for evoking effects of emotional valence during response inhibition.

### 1.2 Aging and response inhibition

Another notable gap in the literature is how typical aging shapes response inhibition for emotional information. This knowledge is particularly relevant in light of well-documented age-related declines in cognitive control and inhibition abilities (Bloemendaal et al., 2016; Gazzaley and D’Esposito, 2007; Smittenaar et al., 2015) alongside age-related improvements in emotional control and regulation (Reed et al., 2014; Reed and Carstensen, 2012) and increasing tendency to appraise ambiguous situations more positively (Mikels and Shuster, 2016). While the stopping process is essentially the same over the adult lifespan, older adults are typically slower and thus less effective at stopping than younger adults (Heathcote and Matzke, 2022; Tsvetanov et al., 2018; Williams et al., 1999).

The cognitive and affective changes with aging are not mutually exclusive. For example, older adults may prioritize accuracy over speed in inhibition tasks (Campbell et al., 2020; Kardos et al., 2020; Williams et al., 1999). Moreover, these factors may intersect such that emotional appraisal and regulation processes are implemented differently between younger and older adults during the initial emotional stimulus perception and detection stages prior to implementing response inhibition (Reed and Carstensen, 2012). For example, older adults demonstrate relative increases in attention to positive information over negative information as compared to younger adults (Carstensen and DeLiema, 2018; Mather and Carstensen, 2003). Greater attention and control over responses to emotional information with aging can, in some cases, yield better emotional response inhibition in older than younger adults. For instance, older adults had fewer false alarms than younger adults on an emotional go/no-go task, and older adults particularly bested younger adults in inhibitory control for emotionally positive stimuli (Waring et al., 2019).

Another limitation of the existing literature of emotional response inhibition is that most investigations have focused solely on the effects of negative valence (versus neutral/non-emotional) on performance in stop trials. Merely a third of the stop-signal tasks described above considered the effects of positive valence (e.g., pleasant images, happy facial expressions) in comparison to negative valence (e.g., unpleasant images, fearful or angry facial expressions), and reports have been inconsistent. Consequently, still little is known about the relatively enhancing or impairing effects of positive compared to negative valence on response inhibition. Taken together, the gaps in knowledge about the effects of aging, emotional valence, and their potential interaction on response inhibition are particularly relevant given well-documented changes in socio-emotional goals and emotional valence processing across the adult lifespan (Carstensen, 2019; Carstensen et al., 1999).

Our 2020 study was the first to compare younger and older adults’ emotional response inhibition during a stop-signal task (Williams et al., 2020). We discovered that the faciliatory effects of task-relevant positive stop-signals (compared to negative stop-signals) were common to both younger and older adults, while positive stop-signals enhanced response inhibition over neutral stop-signals uniquely in older but not younger adults. Moreover, the faciliatory effect of positive over negative stimuli on response inhibition was more pronounced in older than younger adults (effect size *d* = 0.70 vs. *d* = 0.58, respectively; Williams et al., 2020, study 3). That is, age group moderated the influence of emotion on response inhibition. To our knowledge, only one additional study has investigated emotional response inhibition in late life since 2020. Wang et al. (2024) recently assessed how mild memory impairment impacts older adults’ emotional response inhibition. However, as in several studies in younger adults, they assessed the priming effects of exposure to emotional images before go and stop cues, i.e., emotion was irrelevant to the stop-signal task instructions. Response inhibition was poorer in trials that followed the high-arousal (vs low-arousal) negative and positive images, although response inhibition did not differ between task trials following positive, neutral, or negative images. Thus, Wang et al. (2024) identified significant priming (carry-over) effects of emotional arousal, but not valence, on response inhibition (i.e., stop trials) after viewing emotional or neutral images. Notably, results did not differ from older adults with mild memory impairment. However, this study did not advance knowledge of how older adults implement moment-to-moment (i.e., reactive, explicit) stopping for emotionally pleasant and unpleasant information, and no further work has investigated effects of aging or interactions of aging and emotion on response inhibition. Thus, there are still many important open questions about how aging impacts response inhibition for emotional content.

### 1.3 Present study

The present investigation directly builds upon our prior study, which indicated distinct effects of emotion and aging on stopping ability only when focus on emotion was relevant to task instructions (Williams et al., 2020). The purpose of the present study was to identify the impact of typical aging processes on emotional response inhibition. Specifically, we sought to identify whether the ability to stop responses to positive and negative scene images would differ between older and younger adults. To evaluate this question, we developed a novel version of the stop-signal task, the most common task of reactive response inhibition. Amidst frequent go trial responses distinguishing circle and square shapes, an emotional scene image served as an infrequent stop-signal, which cued participants to withdraw responses in-progress (Verbruggen et al., 2019; Verbruggen and Logan, 2009). This design is similar to one we employed previously where facial expression or gender of human faces served as the stop-signal cue (e.g., stop for happy expressions while go for all other expressions; stop for female faces while go for male faces; Williams et al., 2020). The scene images included in the present study are more complex and require more elaborative appraisal (Moors et al., 2013; Scherer and Moors, 2019) than the facial expressions we employed in prior work. This study design was developed to more closely emulate the stopping processes engaged in response to contexts encountered during our affectively rich daily lives, which are significantly more emotionally complex than the simple auditory or visual stop cues employed in standard stop-signal tasks. The present study offers the first investigation where the content of visual scenes has determined whether inhibition should be implemented trial-by-trial.

Building on prior research into the effects of aging and of emotion on reactive response inhibition, we expected an interaction between age group and emotion on response inhibition, such that older adults would show poorer stopping efficiency for unpleasant than pleasant scenes relative to younger adults. We also expected a general effect of aging where older adults would demonstrate less efficient stopping overall compared to younger adults.

## 2 Materials and methods

### 2.1 Participants and enrollment

Thirty-nine younger adults (28 women, 11 men; age *M* (*SD*) = 19.74 (2.53); age range 18–30) and 40 older adults (22 women, 18 men; age *M* (*SD*) = 73.05 (5.22); age range 65–86) were included in analyses. Additional sample demographic information is reported in Table 1. Data from an additional seven enrolled participants were not included in analyses due to: poor adherence to study instructions, such as eating during the study task (*n* = 1 older adult); pronounced tremors observed during the experimental tasks (*n* = 1 older adult); or miss rate above 33% for either pleasant or unpleasant scene images in go trials, suggesting possible difficulty understanding task instructions or atypical interpretation of task stimuli (*n* = 5 older adults).

**Table 1:** Demographic descriptive statistics by age groups.

	Older adults (*N* = 40)	Younger adults (*N* = 39)
Gender		
Male	18 (45%)	11 (28%)
Female	22 (55%)	28 (72%)
Age (years)		
Mean (SD)	73.05 (5.22)	19.74 (2.53)
Education (years)		
Mean (SD)	17.70 (2.72)	13.18 (2.00)
Race		
Black or African American	4 (10%)	4 (10%)
White	36 (90%)	15 (38%)
Asian	0 (0%)	14 (36%)
More than one race	0 (0%)	1 (3%)
Other	0 (0%)	5 (13%)
Ethnicity		
Hispanic	2 (5%)	9 (23%)
Non-Hispanic	38 (95%)	30 (77%)
MMSE		
Mean (SD)	29.40 (0.81)	-

MMSE, Mini Mental State Exam (Folstein et al., 1975).

Saint Louis University Institutional Review Board approved the research protocol, in accordance with the Declaration of Helsinki. Younger adult participants were recruited at Saint Louis University using an online enrollment system (SONA Systems, Bethesda, MD, USA) and flyers posted on campus. Older adult participants were recruited from the St. Louis, MO area via the Washington University School of Medicine Research Participant Registry^2^ and word of mouth. The younger adults recruited online were screened for eligibility with an anonymous questionnaire (Qualtrics, Provo, UT, USA). Younger and older adults recruited via advertising flyers or the Research Participant Registry were screened for eligibility using a short phone interview.

Participants were native English speakers living in the St. Louis, MO region. Younger adults aged 18–30 years and older adults at least age 65 (no upper age limit) were eligible to participate. Exclusion criteria included uncorrected vision or hearing problems; any significant medical conditions that could affect compliance with the protocol (e.g., difficulties speaking, using a pen/pencil to write, using standard computer keyboard and mouse); a history of severe head injury; current life-shortening illness (e.g., cancer); neurological conditions (e.g., seizure disorder, stroke, brain tumor); neurodegenerative disorders (e.g., Parkinson’s, Huntington’s, multiple sclerosis); Autism Spectrum Disorder or Asperger’s syndrome; diagnosis or treatment for anxiety or depression within the past five years; prior or current diagnosis of any other major mental illnesses (e.g., bipolar disorder, schizophrenia, PTSD), use of psychiatric mediations within last five years; a history of alcoholism or substance abuse within the last two years; current use of any central nervous system altering medications (e.g., opiates); current use of memory-enhancing medication; or current symptoms of depression as indicated by Patient Health Questionnaire (PHQ-2) ≥ 3 (Kroenke et al., 2003) at time of screening. Additionally, older adults were screened for possible cognitive impairment, as indicated by a Short Blessed Test score ≥ 5 (Katzman et al., 1983). After enrollment adequate visual acuity was also confirmed in-person using the Snellen eye chart, with additional near-vision screening while seated at the computer.

An additional 13 younger adult participants (6 women, 7 men) and 8 older adult participants (5 women, 3 men) completed a pilot version of the study to iteratively refine the experimental task stimulus set, instructions, and parameters. Pilot participants provided informed consent and conformed to the same inclusion and exclusion criteria, recruitment approaches, and compensation as described, but they were not included in the final sample.

### 2.2 Measures

Participants completed several neuropsychological measures and self-reports of symptoms of depression and anxiety to characterize the sample and to ensure that our older adult sample was cognitively unimpaired. The list of measures with descriptive and inferential statistics between age groups are reported in Supplementary Tables S1, S2. Results of the Mini-Mental State Examination (MMSE; Folstein et al., 1975) indicate that older adults in our sample were cognitively unimpaired [*M* (*SD*) = 29.4 (0.81), range 28–30].

### 2.3 Emotional stop-signal task

#### 2.3.1 Stimuli

**Figure 1:**
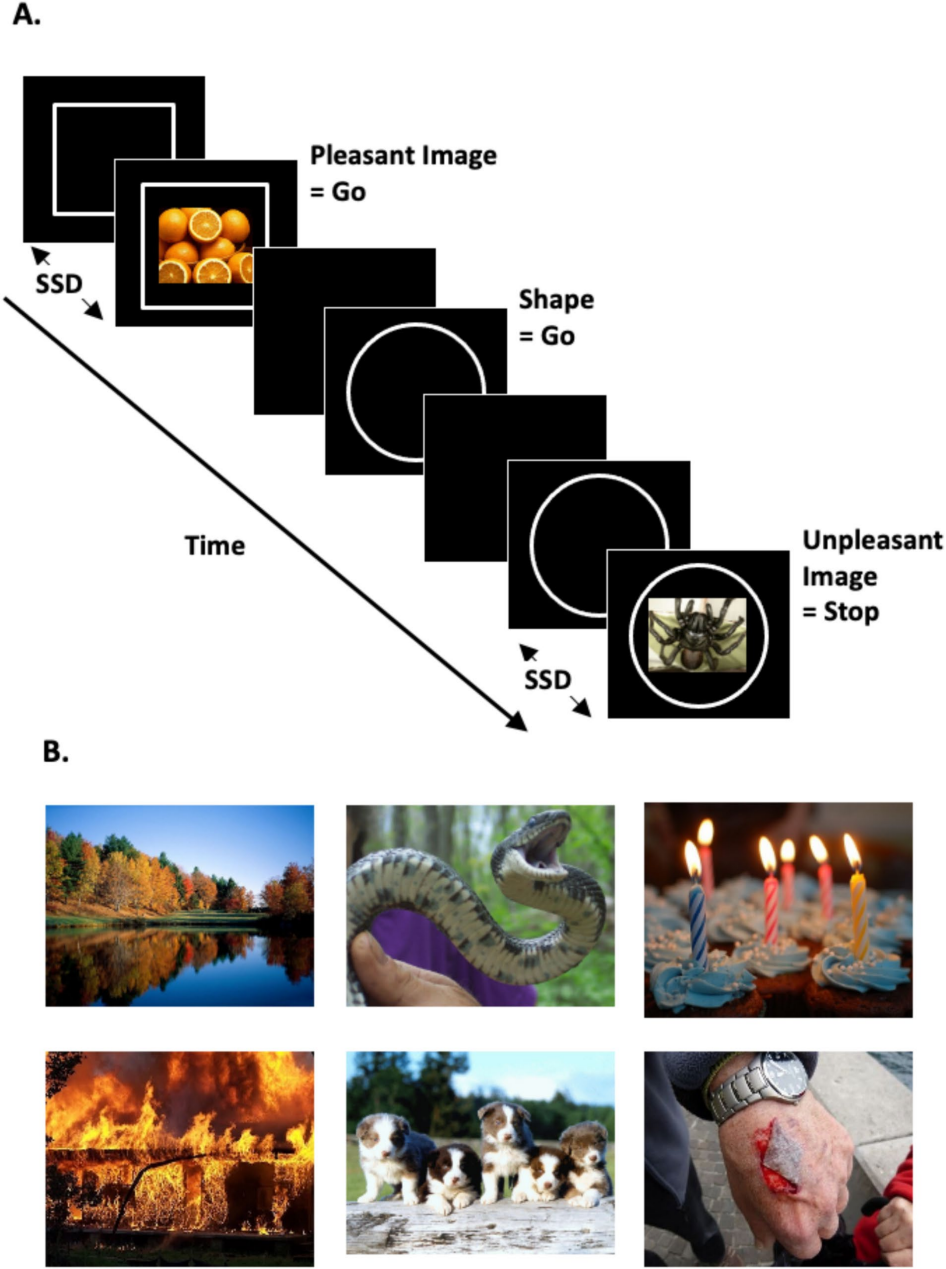
Stop-signal task schematic and scene image examples. **(A)** Stop-signal task design schematic depicting the three trial types within a task block providing instruction to ‘Stop your response when you see an unpleasant image appear’, (L to R): go-image (i.e., fresh oranges = pleasant image), go-shape, and stop trial (i.e., venomous spider = unpleasant image). During go trials (i.e., both go-shape and go-image trials) participants responded to the go-signal (circle or square). During stop trials they were instructed to stop their motor response already in progress toward the go-signal upon onset of a stop-signal, in this example the onset of unpleasant images. The stop-signal followed the go-signal after an adaptive variable-length delay, the stop-signal delay (SSD), which was independent for each stop-signal emotion condition (unpleasant or pleasant scene images) to maintain behavioral performance at approximately 50% correct. **(B)** Example pleasant (p) and unpleasant (u) scene images employed, (L to R): autumn vista (p), threatening snake (u), birthday cupcakes (p), catastrophic fire (u), playful puppies (p), bleeding wound (u). Scene images were selected from the OASIS database (Kurdi et al., 2017; available at www.benedekkurdi.com/#oasis), and the GAPED database (Dan-Glauser and Scherer, 2011; available at https://www.unige.ch/cisa/research/materials-and-online-research/research-material/).

Two hundred thirty-five color scene images were selected from the Open Affective Standardized Image Set (OASIS; Kurdi et al., 2017), with 25 additional scene images taken from the Geneva Affective PicturE Database (GAPED; Dan-Glauser and Scherer, 2011). OASIS is a recent open-access set of affective images normed for both valence and arousal by a more demographically diverse sample than previously published emotional image stimulus sets. The final OASIS plus GAPED stimulus set for the present study was selected through iterative refinement with a pilot participant sample to ensure the content of task images was unambiguous and valence interpretations (i.e., pleasant, unpleasant) were highly consistent with published normative ratings and across participants. The final stimulus set employed 120 ‘pleasant’ and 120 ‘unpleasant’ scene images from OASIS and GAPED sets, with 20 additional unique OASIS scene images included in the practice task (10 each for pleasant and unpleasant images). Examples of unpleasant scene images included: dangerous animals, garbage, explosions, weapons, physical injuries, and feces. Examples of pleasant scene images included: playful baby animals, pastoral scenes, objects typical of celebrations, enjoyable activities, and appetizing foods. Notably, human facial expressions were not visible in any of the images included. See Figure 1 for example stimuli. Image luminance and contrast levels were matched using the SHINE_color toolbox in MATLAB R2019a (Dal Ben, 2023; Willenbockel et al., 2010). The image set reliably represented the assigned valence type, as evidenced by significantly higher published mean pleasantness ratings (Dan-Glauser and Scherer, 2011; Kurdi et al., 2017) for pleasant [*M* (*SD*) = 5.80 (0.41)] versus unpleasant scene images [*M* (*SD*) = 2.39 (0.60); *t*(238) = 51.01, *p* < 0.001, *d* = 6.59]. Additionally, the images set had a moderate mean arousal level, while arousal ratings were higher for unpleasant images [*M* (*SD*) = 4.14 (0.61)] than pleasant images [*M* (*SD*) = 3.72 (0.84); *t*(238) = 4.51, *p* < 0.001, *d* = 0.58]. The full list of images with ratings is included in Supplementary Table S3.

#### 2.3.2 Stop-signal task design

The task parameters were selected to conform with the consensus of recommended best practices for stop-signal task design and protocol (Bissett et al., 2021; Matzke et al., 2018; Verbruggen et al., 2019). The present stop-signal task was modeled off an earlier version implementing human faces as stop-signals, which was originally designed and programmed by Pessoa et al. (2012) and later modified by Williams et al. (2020). The task presented three trial types: go-shape, go-image, and stop trials. In “go-shape trials,” participants identified a shape as a circle or square by pressing the left or right arrow key, respectively. Each go-shape stimulus remained on the screen for a fixed duration of 1,000 ms. Participants were instructed to respond as quickly and accurately as possible on go trials. Occasionally, an emotional image (i.e., pleasant or unpleasant) appeared for 2000 ms within the shape after a variable delay interval (described in more detail below). Before beginning the task, participants were instructed to stop their responses for one image type (e.g., stop your response for pleasant images; “stop trials”) while continuing their responses to the other image type (e.g., continue keypress for unpleasant images; “go-image trials”). The go-signal shapes remained visible for the full duration of stop (and also go-image) trials. A black screen was presented after each go or stop stimulus disappeared in order to maintain an equal trial duration of 3,000 ms across all trial types. Each trial was followed by a black screen ITI with variable duration 900-1100 ms. See Figure 1A for task design schematic. Stop trials were rare (25%) to create a prepotent urge to respond. Participants completed three task blocks with one stop-signal type (e.g., stop for pleasant images), then three blocks of the other stop-signal type (e.g., stop for unpleasant images), with order of the two stop instructions counterbalanced across participants.

The stop-signal delay (i.e., the delay period from the go-shape onset to stop-signal onset within trial) adjusted adaptively in 50 ms increments in response to performance on the preceding stop trial, with a starting delay of 250 ms. For example, if a response was not successfully inhibited, then the stop-signal delay shortened by 50 ms on the next trial to improve the chance of successful inhibition (min 50 ms); if the response was successfully inhibited then the next stop-signal delay lengthened by 50 ms to increase task difficulty (max 950 ms). The dynamic adjustment of the stop-signal delay ensures an approximately 50% success rate for each stop-signal emotion condition, which is necessary to reliably calculate the stop-signal reaction time (SSRT) to measure response inhibition performance (Logan, 1994; Verbruggen et al., 2019). Unique adaptive stop-signal delays were computed separately for pleasant and unpleasant stop conditions for each participant, i.e., the stop-signal delay reset to the starting delay value (250 ms) when the stop-signal instruction changed between blocks three and four. In go-image trials the delay interval between onset of shapes and go-images was yoked to the stop-signal delay of the preceding stop trial, but accuracy on go-image trials did not impact the stop-signal delay for subsequent trials.

There were 480 trials divided among six task blocks. Each 80-trial block comprised 20 stop trials, 40 go-shape trials, and 20 go-image trials (total: 120 stop, 240 go-shape, and 120 go-image trials). Trial types sequence and images sequence were in a predetermined pseudo-randomized order. Each participant saw each scene image one time during the task. The task was programmed using Presentation (Version 23.0; Neurobehavioral Systems, Berkeley, CA, USA).

A significant innovation of the present study over all prior emotional stop-signal task designs is that the scene images employed could indicate either a stop-signal or a signal to continue the motor response underway toward the keypress (i.e., go-signal), depending upon whether the image was pleasant or unpleasant. That is, while the strong majority of trials comprised simple shape discrimination (‘Go’ to circles and squares), the rare onset of scene images could indicate either a stop-signal or an additional type of go-signal. Thus, half of the scene images were stop-signals and half were go-signals, enabling additional exploratory comparisons of responses on stop and go-image trials. This design addresses critiques that the stop-signal is fundamentally different from go-signals in that stop-signals typically have differing relative frequency of presentation (i.e., contextually rarer) and differing attentional and perceptual demands (Wessel, 2025).

### 2.4 Study procedures

Participants completed the protocol in a soundproof booth. All participants provided written informed consent and HIPAA authorization at the time of enrollment. Then participants provided demographic information, and eligibility was reconfirmed. Next, participants completed the neuropsychological and self-report measures. Study data were maintained in REDCap (Harris et al., 2009, 2019).

#### 2.4.1 Stop-signal task

Participants were seated approximately 45 cm from a Dell 24-inch desktop monitor (Round Rock, TX, USA) with 1920 × 1,080 active signal resolution that was projected from a Hewlett-Packard ProBook (Palo Alto, CA, USA) running Microsoft Windows 10 (Redmond, WA, USA) outside the soundproof booth. Participants completed a stop-signal task, inhibiting responses to either pleasant or unpleasant scene images in each block, as described earlier. Image size was standardized as 18 cm wide by 14.5 cm high on-screen. Researchers emphasized that the content or circumstances depicted in the image should be considered when evaluating if it was pleasant/unpleasant, not the skills of the photographer. The importance of responding quickly to go trials and stopping responses on stop trials were emphasized equally in task instructions. Participants performed two short practice blocks—one before the first task block began and another when the instructions changed between blocks three and four—to confirm understanding and adherence to instructions before starting the task. Prior to beginning the recorded task all participants practiced until they were comfortable with instructions and attained sufficient response accuracy. If responses to go-shape or go-image trials were too slow (>1,000 ms) or missed, then a warning message appeared on the screen to further encourage speeded responses (Leotti and Wager, 2010). Twelve younger adults and 13 older adults completed the task while EEG and ECG data were recorded. Electrophysiological data are not reported here.

#### 2.4.2 Post-task feedback survey

After completing the stop-signal task, participants answered a brief self-report questionnaire of their experience completing the task. First, they reported the relative allocation of their total effort the two competing task goals: accurately inhibiting responses on the stop trials and quickly responding on go trials (i.e., effort sums to 100%). They also rated the perceived difficulty of completing the task using a 9-point Likert scale, from 1 = “extremely easy” to 9 = “extremely difficult.” Responses about allocation of effort for two people were excluded from analyses because they misinterpreted the question. Results reported in Supplementary materials.

#### 2.4.3 Task stimuli ratings

Lastly, to aid interpretation of task performance, participants completed a stimulus rating task to ascertain their individual perception of the pleasantness or unpleasantness of each image (Siemer et al., 2007). Participants were instructed to evaluate each of the scene images from the stop-signal task one-by-one as either ‘pleasant’ or ‘unpleasant’ based on their first instinct. Researchers emphasized that participants should evaluate each image based on their own personal interpretation of the pleasantness/unpleasantness of its content rather than the photographer’s skill, in keeping with how they had evaluated the images during the stop-signal task. The ratings task was implemented using MouseTracker (Freeman and Ambady, 2010). In each self-paced trial, an image from the stop-signal task appeared on-screen for 4,000 ms and during this time participants moved the mouse from the bottom-center of the screen to click on one of two response boxes: one labeled ‘UNPLEASANT’ in the upper-left side and the other labeled ‘PLEASANT’ in the upper-right side. All participants practiced the instructions before the recorded task began. On average, participants’ self-reports strongly agreed with the published ratings (Dan-Glauser and Scherer, 2011; Kurdi et al., 2017) both for unpleasant images [younger adults *M* (*SD*) = 99% (0.01), older adults *M* (*SD*) = 96% (0.09)] and pleasant images [younger adults *M* (*SD*) = 98% (0.02), older adults *M* (*SD*) = 99% (0.02)]. In addition to the nearly perfect average concordance of participants’ image ratings with published ratings, we also used the stimuli ratings to compute individual affective bias scores for each participant. In the context of the present design, an affective bias is an individual’s tendency to evaluate images more positively or more negatively than the published ratings, i.e., their proportion of ‘pleasant’ image ratings agreeing with published normative responses minus proportion of ‘unpleasant’ image ratings agreeing with published normative responses. Positive values indicate a more positive bias and negative values indicate a more negative bias, with zero indicating no bias in either direction.

Upon completing the protocol, they were debriefed about the study purposes and compensated for their time. Participants enrolled in the behavioral protocol were compensated with course credit or $20 for their time and effort, and participants who completed protocol with EEG recording received an additional $20.

### 2.5 Analysis approach

All data processing and analyses were computed in RStudio (version 2023.09.1 + 494; Posit Software, Boston, MA, USA).

#### 2.5.1 Computing stop-signal reaction time (SSRT)

SSRT is the primary outcome measure of response inhibition performance in stop-signal tasks. As the time needed for stopping (i.e., not responding) cannot be measured directly, it is best characterized by the imputed value of the SSRT (Congdon et al., 2012; Verbruggen et al., 2019). SSRT was computed using the integration method averaged across blocks (with replacement of go omissions with the maximum allowable go-shape trial response time; Verbruggen et al., 2013). The block-based integration method with replacement of go omissions creates the most reliable and least biased SSRT because it best accounts for 1-participants with strategic response slowing while waiting to see if a stop-signal will appear, and 2-general effects of fatigue across the study. See Verbruggen et al. (2013, 2019) for discussion of this approach as an optimal method for estimating SSRT. To evaluate response inhibition by valence, SSRTs for unpleasant and pleasant images were estimated independently.

In order to uphold the tenets of the independent race model upon which validity of the SSRT relies, we also followed stop-signal task consensus analysis guidelines (Matzke et al., 2018; Verbruggen et al., 2019) and ensured that only the task blocks with valid data were retained for analyses, as follows. Go-shape trial response omissions were rare, *M* (*SD*) = 2.32% (2.11), max. = 9%. All participants had high accuracy (i.e., excluding omissions and incorrect keypress) on go-shape trials, ensuring sufficient alertness and comprehension of instructions, *M* (*SD*) = 96.54% (2.71), min. = 88%. There were no go-shape response times under 150 ms. Within the final participant sample, we excluded from analyses the specific task blocks with go-image accuracy <66% (*n* = 4 older adults), because low go-image accuracy could be attributable to confusion with instructions and/or highly atypical image valence interpretations. As in the go-shape trials, participants demonstrated high accuracy on go-image trials: positive go-images *M* (*SD*) = 96.88% (3.41), min. = 82%, negative go-images *M* (*SD*) = 94.44% (4.82), min. = 77%. We also excluded task blocks with stopping accuracy outside bounds of 25–75% (*n* = 3 older adults) to ensure the adaptive stop-signal delay tracking procedure was performing reliably (Congdon et al., 2012; Verbruggen et al., 2019) and because extreme high or low stopping rates could reflect not only inhibitory control performance but also either confusion with instructions or atypical image valence interpretations. All participants had at least two (of three) task blocks with each stop instruction retained for analyses. After implementing data exclusion criteria, two-tailed independent samples *t*-tests indicated that the quantity of data retained did not differ between younger and older adults [*t*(77) = 0.52, *p* = 0.60, *d* = 0.12; younger adults *M* (*SD*) = 5.87 (0.41), older adults *M* (*SD*) = 5.82 (0.38) task blocks included in analyses].

#### 2.5.2 Statistical analysis plan

A mixed effects analysis of variance (ANOVA) was computed on SSRTs to test for significant interaction of between-subjects factor of age group (2 groups: younger adults, older adults) and within-subjects factor of stop-signal emotion condition (2 emotions: pleasant, unpleasant image).

While SSRT is the typical stop-signal task outcome measure for evaluating response inhibition performance, exploratory analyses considering additional task measures such as the responses on go-image trials offer further context for interpreting the results in this novel stop-signal task. To inform correct response execution we implemented an ANOVA on go-image accuracy with factors of age group and emotion condition (pleasant or unpleasant go-images). Moreover, the stop-signal delay is an adaptive value that increases or decreases the task difficulty to maintain task accuracy around 50%, thus it can be a proxy for stopping accuracy: poorer accuracy yields a shorter stop-signal delay. A parallel ANOVA on stop-signal delays adds further context for interpreting response inhibition performance.

Follow-up *t*-tests were computed as needed to interpret significant interactive effects. Two-tailed paired *t*-tests tested effects between emotion conditions, while 2-tailed independent samples *t*-tests tested effects between age groups. When Levene’s test indicated unequal group variances then Welch’s two-sample *t*-test was applied. Effect sizes were computed for all analyses: Cohen’s *d* for *t*-tests and $\eta_{p}^{2}$ for F-tests. All task analyses were repeated independently each with affective bias score and self-reported anxiety as covariates, as reported in Supplementary materials and summarized in Discussion section.

#### 2.5.3 Power analysis

An *a priori* power analysis for sample size needed to detect the hypothesized significant effects was based on a mixed ANOVA with an interaction of between-subjects factor of age group and within-subjects factor of stop-signal emotion condition. The power analysis for a small-to-medium effect size (*f* = 0.2; based on Williams et al., 2020, study 3), 0.05 alpha error probability (2-tailed), and 90% power determined that a total sample of *N* = 68 would yield sufficient power to detect significant interactive effects (G*Power 3; Faul et al., 2007). As described earlier, the final sample included in analyses was *N* = 79 (younger adults *n* = 39, older adults *n* = 40), confirming that analyses were sufficiently well-powered to detect the hypothesized effects.

## 3 Results

While response accuracy on go-shape trials nearly reached ceiling level, *M* (*SD*) = 96.54% (2.71), younger adults had higher accuracy than older adults, *t*(74.23) = 2.07, *p* = 0.04, *d* = 0.46; younger adults *M* (*SD*) = 97.16% (2.34), older adults *M* (*SD*) = 95.93% (2.92). Response omissions on go-shape trials approached floor level [*M* (*SD*) = 2.32% (2.11)] and did not differ between younger and older adults [*t*(77) = 1.90, *p* = 0.06, *d* = 0.43]. See Table 2 for go-shape trials percentage accuracy and omissions reported by age group. Younger adults’ correct responses to go-shape trials were significantly faster than older adults’ responses, *t*(61.10) = 3.14, *p* = 0.003, *d* = 0.71; younger adults *M* (*SD*) = 732.54 ms (111.44), older adults *M* (*SD*) = 797.28 ms (65.41). The mean response times for failed stop trials for unpleasant images and for pleasant images were each faster than the mean response times for each type of go trial (i.e., go-shape, unpleasant go-images, and pleasant go-images; all *t*s(78) > 3.45, all *p*s < 0.001, all *d*s > 0.38; for full report see Supplementary Table S4), upholding the tenets of the independent race model of the stop-signal task (Verbruggen and Logan, 2009). See Table 2 for response times reported by age group and trial type. The inhibition rate (i.e., successfully stopping) for unpleasant and pleasant images were 55.9% and 54.5%, respectively, indicating that the stop-signal delay adaptive tracking was effective at producing roughly equal proportions of successful and unsuccessful stop trials.

**Table 2:** Stop-signal task descriptive statistics.

	Older adults (*N* = 40)	Younger adults (*N* = 39)
	*M* (SD)	*M* (SD)
Unpleasant Stop images inhibition (%)	56.38 (3.00)	55.39 (3.81)
Pleasant Stop images inhibition (%)	54.96 (3.69)	54.10 (3.43)
Stop Signal Delay unpleasant images (ms)	506.96 (109.85)	483.03 (129.87)
Stop Signal Delay pleasant images (ms)	518.31 (88.82)	481.54 (140.33)
SSRT unpleasant images (ms)	250.28 (61.41)	208.60 (51.14)
SSRT pleasant images (ms)	257.07 (54.30)	222.27 (43.10)
Go-shape trials accuracy (%)	95.93 (2.92)	97.16 (2.34)
Go-shape trials response omissions (%)	2.76 (2.08)	1.87 (2.08)
Go-shape trials RT (ms)	797.28 (65.41)	732.54 (111.44)
Unpleasant go-image trials RT (ms)	1019.2 (88.81)	894.02 (125.73)
Pleasant go-image trials RT (ms)	957.02 (96.23)	840.67 (130.69)
Failed stop to unpleasant images RT (ms)	771.11 (84.88)	687.10 (103.38)
Failed stop to pleasant images RT (ms)	750.29 (83.61)	670.21 (111.33)

The stop-signal delay adaptive tracking procedure worked properly, as indicated by successful inhibition rates around 50%. SSRTs did not differ between pleasant and unpleasant images, but were longer for older than younger adults. Go-shape trials response omission rate was near floor level and accuracy approached ceiling level. Failed stop RTs are significantly shorter than go-shape and go-image trial RTs, indicating that the race model of stop-signal task is upheld. SSRT = Stop-signal reaction time. RT, response time.

**Figure 2:**
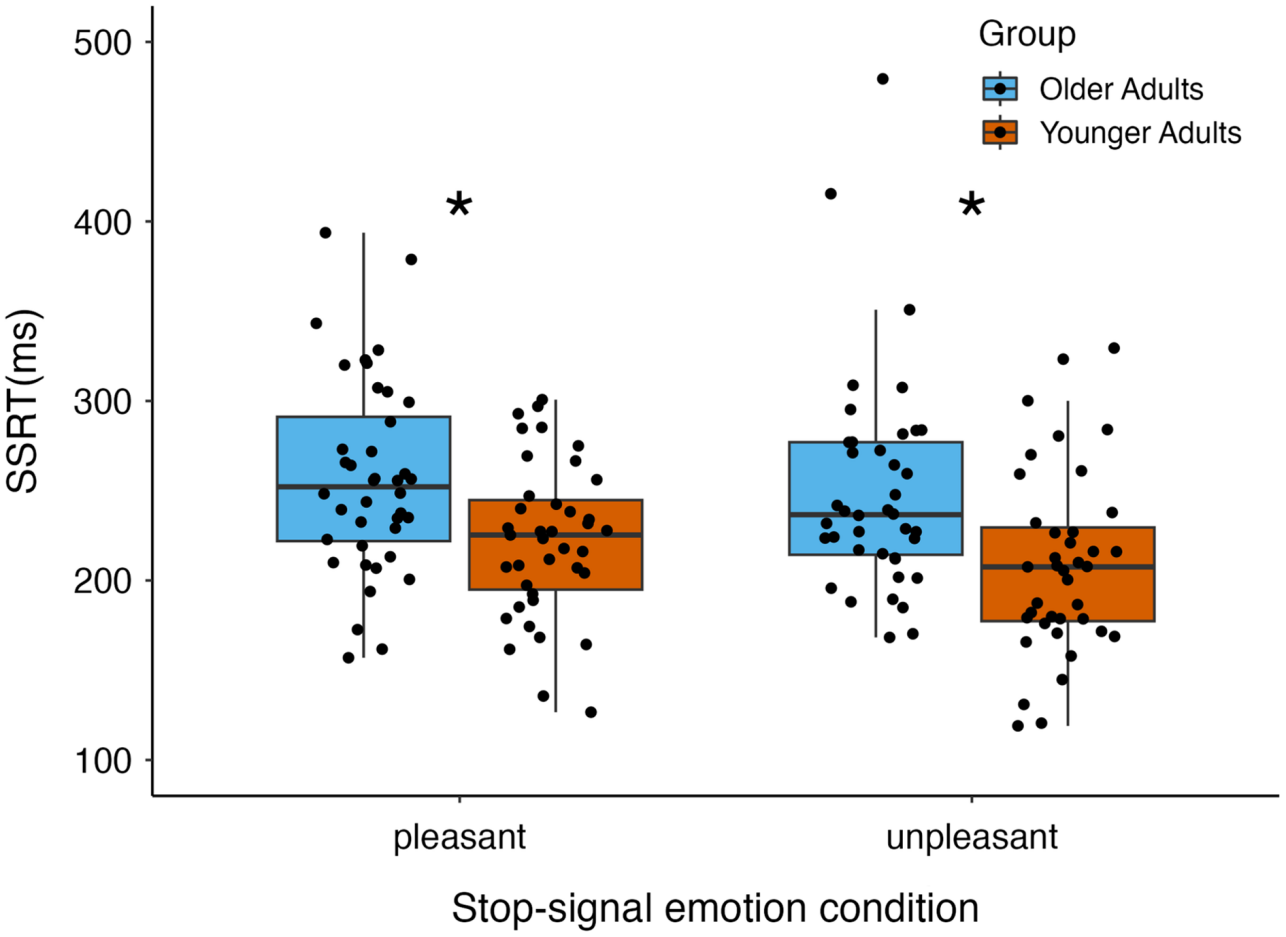
Stop-signal reaction time results by age group and emotion condition. Older adults had significantly longer stop-signal reaction times (SSRTs) than younger adults. SSRTs did not differ when stopping for pleasant versus unpleasant scene images, and age group did not interact with emotion condition. *p = 0.002.

The mixed-effects ANOVA of the effects of age and stop-signal emotion condition on SSRTs showed a main effect of age group [*F*(1,77) = 14.06, *p* < 0.001 $\eta_{p}^{2}$= 0.15] because SSRTs were significantly longer for older than younger adults, indicating less efficient response inhibition overall in older adults (see Figure 2). There was no main effect of emotion [*F*(1,77) = 2.73, *p* = 0.10, $\eta_{p}^{2}$ = 0.03] or interaction between age group and emotion on SSRTs [*F*(1,77) < 0.31, *p* = 0.58, $\eta_{p}^{2}$ = 0.004]. See Table 2 for mean SSRTs reported by age group and emotion.

**Figure 3:**
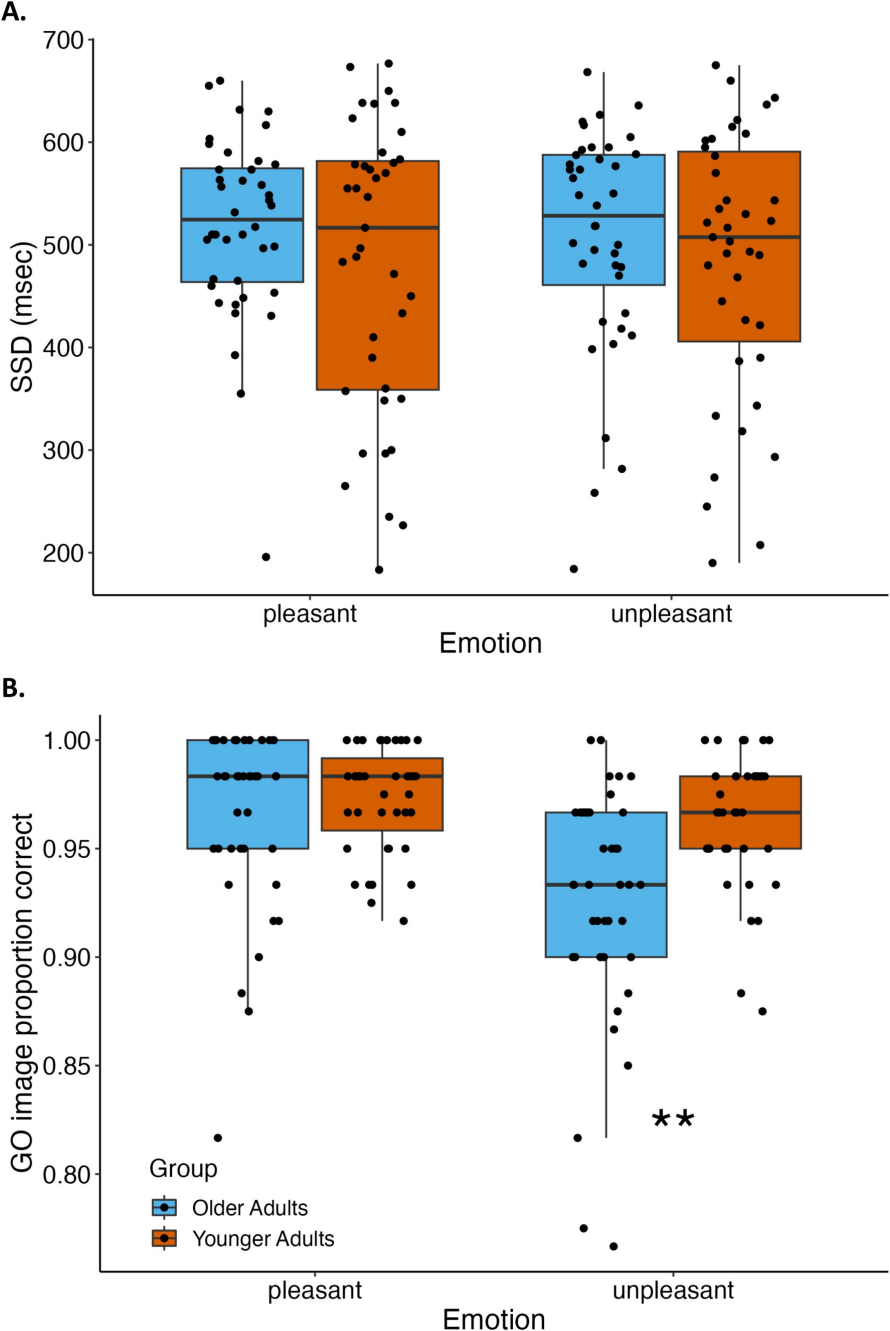
Stop-signal delays and go-image accuracy by age group and emotion condition. **(A)** Stop-signal delay (SSD), which adaptively adjusts with accuracy on stop trials, did not differ between younger and older adults or between pleasant and unpleasant scene images. **(B)** Accuracy of correctly completing responses on go-image trials was significantly greater for younger than older adults, and also greater for pleasant than unpleasant images. Effects of age group and emotion condition interacted because older adults had lower accuracy than younger adults when responding to unpleasant go-images, but younger and older adults’ accuracy did not differ when responding to pleasant go-images. **p < 0.001.

Exploratory analyses on stop-signal delays indicated no significant main effect of age group [*F*(1,77) = 1.60, *p* = 0.21, $\eta_{p}^{2}$= 0.02] or emotion [*F*(1,77) = 0.18, *p* = 0.67, $\eta_{p}^{2}$= 0.002] or interaction of age group and emotion [*F*(1,77) = 0.30, *p* = 0.59, $\eta_{p}^{2}$= 0.004]. In contrast, emotion and age group had marked effects on go-image accuracy. See Figure 3 for depiction of stop-signal delays and go accuracy by emotion and age group. There was a main effect of age group [*F*(1,77) = 10.42, *p* = 0.002, $\eta_{p}^{2}$= 0.12] because go-image response accuracy was higher for younger than older adults. There was also a main effect of emotion [*F*(1,77) = 22.60, *p* < 0.0001, $\eta_{p}^{2}$= 0.23] because go-image accuracy was higher for pleasant than unpleasant images. These main effects were qualified by an interaction between age group and emotion [*F*(1,77) = 9.04, *p* = 0.004, $\eta_{p}^{2}$= 0.11] revealing that younger adults had higher accuracy than older adults only when responding to unpleasant go-images [*t*(62.15) = 3.92, *p* < 0.001, *d* = 0.88]; there was no significant difference between younger and older adults’ response accuracy for pleasant go-images [*t*(77) = 1.05, *p* = 0.30, *d* = 0.24]. See Table 2 for all stop-signal task outcome measures reported by age group and emotion.

## 4 Discussion

The current study aimed to identify the impacts of emotion and typical aging on reactive response inhibition. To do so, younger and older adults completed a novel stop-signal task design employing a large set of emotionally pleasant and unpleasant scene images as explicit stop cues. This design was developed to better model the stopping processes engaged during our affectively rich daily lives, which are more complex than the stopping processes elicited by the simple auditory tones or color change cues used in standard stop-signal tasks. Results revealed that older adults have less efficient stopping ability than younger adults overall when emotion is the explicit focus of the response inhibition goals. However, response inhibition does not significantly differ between pleasant and unpleasant information for either age group. These insights substantively expand the limited literature of how aging impacts inhibitory control in emotional contexts. Notably, this study is only the second to date directly comparing the stopping efficiency of younger and older adults when emotion is explicitly task-relevant to the goals of implementing inhibition (Williams et al., 2020).

### 4.1 Implementing inhibition for emotional information

While a growing literature has investigated the implicit and explicit effects of emotion on response inhibition in younger adults, prior studies have generally treated emotion as merely incidental to implementing inhibition. Consequently, our earlier study provided the primary basis for deriving expectations about the effects of aging on task-relevant emotional response inhibition in the current study. Using a simpler variety of an explicit emotional stop-signal task, we previously observed significantly less efficient stopping for negative (fearful) facial expressions than positive (happy) ones, yet no mean difference in response efficiency between younger and older adults (Williams et al., 2020, study 3). We concluded then that emotional information impacts response inhibition similarly in both age groups when attentional focus on the emotional attributes of stimuli is necessary to successfully complete the task. Other investigators have drawn similar conclusions regarding task-relevance of attention to emotion as a significant factor for evoking effects of emotion on response inhibition in the context of go/no-go tasks in younger adults (Calbi et al., 2022; Mancini et al., 2022). Given the similarity in task instructions between the current and prior study–both focused attention on the emotional attributes of stimuli (i.e., emotion as task-relevant)–we anticipated similar patterns of results: impairing effects of negative (vs positive) stimuli on stopping efficiency, with no significant effects of aging. However, results did not demonstrate the expected effects. We believe the most plausible explanation for the distinction in study outcomes lies in the greater emotional appraisal demands necessary to determine the correct behavioral response (i.e., stop or go) to the more complex affective stimuli used in the present study than the prior design. Although the affective attributes of the stop-signal were task-relevant in both the current and prior designs, the content of the stop-cue was significantly more varied in the current study. While we employed the three facial expressions (happy, neutral, fearful) in Williams et al. (2020), the present study required participants to identify and appraise a highly varied set of emotionally salient scene images. Images depicted a wide range of content, including pastoral landscapes, gruesome physical injuries, playful puppies and kittens, dangerous animals, feces, and appetizing foods. The broader stimulus set likely imposed greater demands for visual recognition and emotional appraisal than when distinguishing between three types of facial expressions.

Another possible explanation for distinctions between results of Williams et al. (2020) and the present study is that faces and scene images are processed in qualitatively different ways. Facial expressions are important social cues that convey knowledge about the present environment to shape behavior and affect (Hess et al., 2009). Moreover, faces and scenes are processed in distinct brain regions (Downing et al., 2006) and may engage differing neural connectivity with brain regions supporting response inhibition (Ocklenburg et al., 2013). Furthermore, older and younger adults have different socio-emotional goals (Carstensen et al., 1999; Carstensen and DeLiema, 2018) and attend differently to positive and negative faces (Mather and Carstensen, 2003), both of which may promote more pronounced age differences when the stop-signals are faces compared to non-social scenes.

The presentation of emotional images overlaid on the go-signals commanded appraisal of their pleasantness or unpleasantness while a motor response was already in progress, akin to a complex or selective stopping task (Bissett and Logan, 2014; Colonius and Diederich, 2023; Wessel and Aron, 2014). Unlike typical stop-signal tasks, where the onset of the stop-cue (e.g., an auditory tone or color cue) has a static and straightforward meaning (Bissett and Logan, 2014; Wessel and Aron, 2014), the current emotional stop-signal task design required a more deliberate and subjective identification and subsequent appraisal process (Luther et al., 2023; Moors et al., 2013; Scherer and Moors, 2019; Schupp et al., 2006). This design increased the cognitive demands of the task because once the prepotent impulse to respond (i.e., “go”) was established across all trials, the rare presentation of emotional scene images necessitated a decision based on rapid interpretation (i.e., What is the content of this image?) and appraisal (i.e., Is the content of this image pleasant or unpleasant?) to determine the desired course of action: either continuing or stopping the response already underway (Siemer et al., 2007).

It is also important to emphasize that the novel stop-signal task developed for the present study did not merely convert a stop-signal task into a go/no-go task. Reactively stopping an action already underway, as the present task required, is a cognitive process distinct from proactively not initiating a response (Littman and Takács, 2017; Meyer and Bucci, 2016; Raud et al., 2020). Additionally, the timing of the stop-signal onset relies on the adaptive stop-signal delay (ensuring that accuracy is maintained around 50%), which is a critical distinction from go/no-go task designs (Wessel, 2025). Lastly, the very small effect sizes (all $\eta_{p}^{2}$< 0.04) of the null main and interactive effects of emotion on response inhibition should be appreciated in the context of robust analyses well-powered to detect significant effects. Thus, the current study effectively advances knowledge of how reactive response inhibition is implemented for emotional contexts.

Decades of empirical evidence suggest that canceling a variety of motor actions (e.g., arm, hand, and eye movements), speech, and thoughts occurs on similar timescales, and relies on a common domain-general control process irrespective of the modality of behavior (Apšvalka et al., 2022; Hannah and Aron, 2021; Logan, 1994; Logan and Cowan, 1984; Wessel and Anderson, 2024). Moreover, simple and complex stop-signal tasks employ the same core brain networks (Wessel and Aron, 2014). Non-selective inhibition employed in standard stop-signal tasks recruits a direct (i.e., hyperdirect) ventral prefrontal-subthalamic nucleus pathway for general motor suppression, while selective stopping recruits an indirect pathway that engages these areas plus a variety of additional brain regions (Chen et al., 2020; Wessel and Anderson, 2024). Specifically, in the context of the present design, it is likely that a variety of prefrontal and subcortical regions supporting emotional appraisal and regulation are engaged when the pleasant and unpleasant images appear (Denny et al., 2023; Luther et al., 2023). These regions interact with the motor control regions typically engaged during inhibitory control (Apšvalka et al., 2022; Hannah and Aron, 2021; Schmidt and Berke, 2017). However, whether simple versus complex variations of emotional stop-signal tasks, or emotional versus non-emotional complex stopping tasks, share the same neural mechanisms remains to be tested.

### 4.2 Aging and emotion processing

Although speculative, it is plausible that the rapid and highly constrained task demands in the present study eliminate the significant effects of emotion that younger and older adults both evidenced during the simpler version of an emotional stop-signal task (Williams et al., 2020, study 3). As noted earlier, the current design resembles a complex or selective stopping task (Bissett and Logan, 2014; Wessel and Aron, 2014). Under these conditions, older adults’ habitual or automatic emotion processing and regulation are limited both by the task pace and instructions, compared to less-constrained designs (Reed et al., 2014). The more evaluative, appraisal-based selective stopping design may explain why the significant emotion effects that younger and older adults displayed when stopping for one of three facial expressions (Williams et al., 2020) were not observed in the present design. With respect to the rapid pace and constrained demands of the task, it is also intriguing to note that there were no main or interactive effects of age on the stop-signal delay, which indexes stopping accuracy. This finding indicates that, when given slightly more time, older adults can implement reactive stopping for emotional scene images with similar accuracy as younger adults.

Another notable feature of this study design is that we collected participants’ personal interpretations of each of the 240 scene images (pleasant, unpleasant) presented in the stop-signal task. These individual image ratings enabled computing an affective bias score for each person. Affective bias scores allowed us to evaluate: 1- whether our participants’ interpretations of the images were more positively or negatively biased than either published normative ratings or our local pilot study sample, and 2- whether there were significant age differences in image interpretation. These considerations have implications for the reliability of the stop-signal delay’s adaptive tracking of inhibition accuracy, which assumes that study participants’ image interpretations concur with published ratings. Critically, the interpretation of stop- and go-image trial responses did not change when including affective bias scores as a covariate in analyses; all reported effects were replicated even when accounting for the possibility of atypical image interpretation (e.g., a tendency to perceive the scene images more positively or negatively than normative ratings; results reported in Supplementary materials).

In light of significantly higher self-reported anxiety (STAI; Spielberger, 1983) in younger compared to older adults in the present sample (reported in Supplementary materials), we also evaluated whether anxiety significantly impacted task performance. Literature suggests that anxiety can bias sustained attention toward threat-related information (Clauss et al., 2022) and that anxious arousal reduces inhibitory control (Pacheco-Unguetti et al., 2012; Roxburgh et al., 2022), possibly by consuming mental resources needed for effective inhibitory control (Eysenck et al., 2007; Pessoa et al., 2012, study 2). However, including anxiety as a covariate in analyses of the present stop-signal task did not change the interpretation of results. All reported effects were replicated after accounting for the possibility of a significant influence of state or trait level anxiety (reported in Supplementary materials). These additional analyses evaluating the possibilities of affective bias or anxiety effects strengthen our confidence in the validity of the study’s conclusions. Thus, we can discount the possibility that the reported effects were driven by significant systematic age-differences in image interpretation or self-reported anxiety.

### 4.3 Comparing stop and go processes

A further innovation of this stop-signal task design is that in addition to measuring stopping efficiency, it also enabled exploratory analyses of responses executed for go-images, as the occasional images presented could signify either ‘go’ or ‘stop’ cues depending upon the instructions for the respective task block. For example, when instructed to stop for pleasant images, one must still execute the ‘go’ keypress for unpleasant images, and vice versa. This design allowed us to investigate whether emotional valence influenced correctly executed responses (go-image trials) similarly to its impact on inhibited responses (stop trials). Critically, if emotional valence impacted both processes equivalently, then we would expect to observe similar patterns of main and interactive effects of emotion across ‘stop’ and ‘go-image’ trials. We identified that although emotion did not significantly influence stopping efficiency (SSRTs) or stopping accuracy (stop-signal delay), a different pattern emerged for correctly executed responses: go-image responses were significantly impacted by emotion, aging, and their interactive effects, as discussed in the following section.

#### 4.3.1 Insights into aging and emotion processing

The differing results for response execution versus response inhibition for scene images advances basic understanding of how emotion influences response tendencies, highlighting that emotion does not exert uniform effects during response execution and inhibition across the adult lifespan (Waring et al., 2019). We observed that pleasant and unpleasant images differently impact successful response execution in early versus later adulthood, while the effects of emotional valence on response inhibition are relatively stable with aging. The observed main and interactive effects of emotion (and age group) on go-image trials accuracy, contrasted with the absence of such effects in stop trials performance (i.e., both stopping efficiency and accuracy), suggest that older adults can effectively adjust their response strategies when approaching emotional stimuli. These findings align broadly with motivational theories underlying age-related positivity effects (Carstensen, 2019; Carstensen and DeLiema, 2018). That is, the present study suggests that older adults may approach and engage less with unpleasant scenarios than younger adults, while both age groups engage with positive scenarios with comparable accuracy. Importantly, while older adults exhibit poorer response inhibition than younger adults overall, inhibition was comparable across pleasant and unpleasant contexts within each age group. These effects highlight that although aging impacts overall inhibitory control, effects of emotional context on response inhibition appear consistent across the adult lifespan.

#### 4.3.2 Basic insights into response inhibition

The distinct effects of emotional images during stop versus go-image trials advances broader understanding of how the brain implements response inhibition. Neurophysiological evidence from rodent basal ganglia recordings suggests that response inhibition is not a single, uniform ‘stop’ process, but instead is implemented through a two-stage ‘pause-then-cancel’ process (Schmidt et al., 2013; Schmidt and Berke, 2017). According to this model, all images in the present design would initially trigger a pause process, while only images matching the stop instruction would then proceed to full cancellation of the response. In contrast, the prepotent go responses to shapes would not initiate a pause process at all. This framework helps explain the slower responses observed for go-images compared to shapes. When scene images appear—whether serving as stop- or go-signals—their high salience and complexity likely initiate a ‘pause’ process for additional evaluative and appraisal processing (Moors et al., 2013; Scherer and Moors, 2019) prior to selectively applying a full cancellation (i.e., only for trials matching the stop-signal; Diesburg and Wessel, 2021; Wessel, 2025). Future work contrasting younger and older adults’ neural signatures of response inhibition (cancellation) versus execution for emotional stimuli will offer further clarity about these possibilities.

### 4.4 Strengths and limitations of the study

The greater variety and complexity of the stop signals employed in the present study substantially enhances the ecological validity of the design over most prior response inhibition tasks, better emulating the complex behavioral control decisions implemented in daily life (Hannah and Aron, 2021). Standard stop-signal tasks model a scenario of responding to unambiguous and affectively-neutral stimuli, yet beyond canonical examples of traffic lights or sirens, there are very few instances in everyday life where we inhibit or complete behaviors in response to explicit cues that lack affective salience. In daily living, much of our inhibition is implemented to align our thoughts and actions with affective goals, such as avoiding frightening and dangerous situations (e.g., avoiding traffic or broken glass on the street), maintaining social appropriateness (e.g., minimizing embarrassment), avoiding disgust from unsanitary conditions (e.g., avoiding stepping on pet waste or eating spoiled food), and mitigating sadness or anxiety by reducing emotionally maladaptive mindsets (e.g., rumination). Empirical evidence increasingly demonstrates that inhibition of actions and thoughts rely on the same core cognitive and neural resources (Apšvalka et al., 2022; Castiglione et al., 2019; Wessel and Anderson, 2024). Thus, the present design advances the field by offering a more representative model of how individuals rapidly appraise and decide whether to engage or avoid emotionally salient information, and how normative aging impacts these processes. We extended the traditional stop-signal task by implementing stopping decisions based on appraisal of more naturalistic and complex visual images, rather than the auditory tones or simple stimulus color changes that cue stopping in standard tasks. The present study aimed to more closely model the inhibitory processes implemented in real-world scenarios, albeit still within a laboratory environment. Importantly, the paradigm achieves the critical validity checks of the standard stop-signal task and its key outcome measure, the SSRT (Bissett et al., 2021; Matzke et al., 2018; Verbruggen et al., 2019), as affirmed in the methods and results. Thus, this novel stop-signal task design is valid and robust while achieving greater ecological validity.

While the novel study design presented here has many strengths, there are also a few limitations that present opportunities for future investigation. Most notably, we did not include neutral scene images in the present study for two primary reasons, 1-the difficulty of rapidly discriminating neutral images from either pleasant or unpleasant images during the time constraints of the task trials, and 2-the consideration of limiting participant time burden, as adding a third stimulus type would substantially lengthen the task duration. Consequently, our conclusions are limited to comparing response inhibition between pleasant and unpleasant stimuli, without a neutral non-arousing baseline for contrast. Additionally, the need to limit participant time burden also prevented including a conventional stop-signal task to compare within-person performance between complex and simple stopping demands. Recording eye-tracking or electrophysiology (EEG, ERP) in future studies will offer high temporal-resolution evidence of the eye movements or brain responses corresponding with the behavior observed in the present study. Although we adjusted the low-level visual properties of the image stimulus set to match luminance and contrast levels (SHINE toolbox; Dal Ben, 2023; Willenbockel et al., 2010), it is possible that there are remaining differences in other perceptual features across the stimulus set.

While we screened potential participants for diagnosed mental illness and indication of currently elevated anxiety or depression before enrollment, the final participant sample exhibited sporadic cases of subclinical anxiety and depression, representative of the broader population. In particular, younger adult participants reported higher average anxiety than the older adult participants. Although follow-up analyses indicated that anxiety did not significantly impact task performance in the present study (reported in Supplementary materials), an intriguing area of future research is whether emotional response inhibition in this novel stop-signal task design will differ in individuals with clinically diagnosed anxiety disorders (Clauss et al., 2022).

## 5 Conclusion

This is the first study to compare how younger and older adults implement response inhibition in direct response to pleasant and unpleasant scenes. We developed a novel version of the stop-signal task to enhance ecological validity over prior stop-signal tasks and more closely model how adults implement stopping in complex, emotionally arousing contexts. Results show that aging reduces response inhibition overall, and age-related changes are not selective to positive or negative emotional valence. In other words, older adults exhibit similar inhibitory control for complex pleasant versus unpleasant information, although less efficiently than younger adults.

When environmental demands require rapid evaluation and appraisal of complex affectively salient information in order to determine the appropriate course of action, then inhibition is comparable across emotionally pleasant and unpleasant information for both younger and older adults. Exploratory analyses permitted additional insights into the distinction between response tendencies for completing versus inhibiting actions. These analyses revealed that positive valence (versus negative) uniquely benefits executing responses but not inhibiting responses in emotional visual contexts. This study offers the first evidence of the effects of aging and emotional valence on response inhibition for complex, emotionally salient information. In doing so, the present study contributes a more comprehensive picture of how younger and older adults implement inhibitory control in daily living.

## Data Availability

The datasets presented in this study can be found in online repositories. The study data can be found at: Open Science Framework (OSF), https://osf.io/haety/.

## Footnotes

^1^There are also several ways to compute the primary outcome measure of stop-signal tasks, which may be another source of variability among response inhibition literature (Coccaro et al., 2024; Verbruggen et al., 2019). It is unclear if the choice of computation approach may yield different interpretations regarding stopping for negative versus positive stimuli. However, examination of this question is beyond the scope of the current study.

^2^ https://sites.wustl.edu/wuvfh/

## Author’s note

Preliminary results were presented previously at the annual meetings of the Association for Psychological Science, San Francisco, CA (May 2024) and Midwestern Psychological Association, Chicago, IL (April 2024).
