# Supplementary material for: Effects of aging and valence on emotional response inhibition: conclusions from a novel stop-signal task
Source: Front Psychol. 2025 Apr 9;16:1568492. doi: 10.3389/fpsyg.2025.1568492 (PMC12020514; doi:10.3389/fpsyg.2025.1568492)
Supplement: Supplementary file 1 [file Data_Sheet_1.pdf]

Supplemental Materials

Effects of aging and valence on emotional response inhibition: Conclusions from a novel stop-signal task

Jill D. Waring & Stephanie N. Hartling

Methods

Measures

Participants completed measures of executive functioning including the Delis-Kaplan Executive Function System (D-KEFS) Verbal Fluency Test (3 conditions; Delis, Kaplan, & Kramer, 2001) and the Trail Making Test parts A and B (Reitan, 1958). See **Table S1** for descriptive and inferential statistics on these measures by age group. Older adults also completed the Mini-Mental State Examination (MMSE; Folstein, Folstein, & McHugh, 1975) to identify cognitive impairment, as reported in main text.

Table S1: Neuropsychological Testing Descriptive Statistics and Comparisons Between Age Groups

	group	Mean	SD	Min	Max	F	*p*	η_p^2
	**D-KEFS Verbal Fluency**							
Letter Fluency: FAS	OA	47.75	10.22	32	78	17.15	**< .001**	0.18
	YA	38.41	9.82	18	57			
Category Fluency: Animals & Boys' Names	OA	42.98	7.53	29	61	1.07	0.30	0.01
	YA	41.23	7.46	30	61			
Category Switching: Fruits/Furniture	OA	13.68	1.98	10	18	0.11	0.74	0.00
	YA	13.87	3.09	6	24			
	**Trail Making Test**							
Part A (sec)	OA	32.75	8.33	19.54	59.14	44.41	**< .001**	0.37
	YA	21.20	6.99	11.91	47.22			
Part B (sec)	OA	78.12	27.06	41.91	156.22	21.86	**< .001**	0.22
	YA	54.10	16.85	25.00	97.50			
	**Cognitive Status**							
MMSE	OA-only	29.40	0.81	28	30	-	-	-

Notes: *OA = older adults, n = 40; YA = younger adults, n = 39. MMSE = Mini-mental state exam. Significant effects indicated in bold font. Older Adults had higher verbal fluency to letters than younger adults, while younger adults were faster than older adults on Trail Making Test parts A and B. Notably, performance did not differ between groups on category fluency or category switching. Time for Trail Making Test part B was missing for one younger adult due to experimenter recording error. All references provided in supplemental Methods text.*

Additional self-report measures included the Spielberger State-Trait Anxiety Inventory (STAI; Spielberger and Gorsuch, 1983), an anxiety inventory (older adults: Geriatric Anxiety Inventory (GAI; Pachana et al., 2007); younger adults: Beck Anxiety Inventory (BAI; Beck & Steer, 1993)), and a depression inventory (older adults: Geriatric Depression Scale 30-item version (GDS; Yesavage et al., 1983); younger adults: Beck Depression Inventory (BDI; Beck et al., 1961)). See **Table S2** for descriptive statistics for these measures.

Table S2: Self-Report Measures Descriptive Statistics by Age Group

	Mean	SD	Min	Max
**Older Adults**				
Geriatric Depression Scale	2.73	3.50	0	17
Geriatric Anxiety Inventory	0.83	2.36	0	13
STAI State	25.38	6.82	20	51
STAI Trait	27.68	7.69	20	65
**Younger Adults**				
Beck Depression Inventory	4.79	4.45	0	20
Beck Anxiety Inventory	7.92	8.64	0	32
STAI State	45.44	6.23	31	60
STAI Trait	35.82	10.64	21	62

Notes: *Older adults n = 40 and younger adults n = 39 for all measures. Twenty is the lowest score possible on STAI state and trait subscales. Younger adults had significantly higher mean STAI state and trait anxiety scores than older adults (p's < .001, d's > 0.86; full results reported below). All references provided in supplemental Methods text.*

Stop-signal task stimuli

The full list of task stimuli including the valence and arousal ratings published in OASIS (Kurdi et al., 2017) and GAPED (Dan-Glauser & Scherer, 2011) are reported in **Table S3** (see separate spreadsheet). Note that the GAPED database valence and arousal ratings were originally collected using a 100-point scale, and thus were rescaled to a 7-point scale for statistical comparisons to OASIS ratings in the present study. Table S3 reports the rescaled GAPED ratings.

Results & Discussion

Confirming tenets of the independent race model of the stop signal task

The mean response times for failed stop to unpleasant and pleasant image stop-signals were each significantly faster than the mean response times for each type of go trial (i.e., go-shape, unpleasant go-image, pleasant go-image), as reported in **Table S4.** This pattern of effects upholds the tenets of the independent race model of the stop-signal task (Verbruggen et al., 2019).

Table S4: Comparison of stop and go trial response times by trial type

	$t(78)$	p	d
failed stop for unpleasant images			
go-shape	4.02	.0001	0.45
unpleasant go-image	19.71	< .0001	2.22
pleasant go-image	21.31	< .0001	2.40
failed stop for pleasant images			
go-shape	6.05	< .0001	0.68
unpleasant go-image	23.95	< .0001	2.69
pleasant go-image	17.60	< .0001	1.98

Note: *See Table 2 for response time means and standard deviation.*

Considering individual affective biases did not change interpretation of results

As described in the main text, gathering individual participants' categorization of each stop-signal task image as pleasant or unpleasant after completing the task allowed us to compute affective bias scores for each person. Results of ANOVAs on stop-signal task performance that were reported in the main text did not change when including affective bias as a covariate in analyses, as reported below. All significant effects remained significant and null effects remained so after testing the possibility that a bias to interpret task images either as more pleasant or as more unpleasant than published ratings would significantly impact stop-signal task performance.

The mixed-effects ANOVA of the effects of age and emotion on SSRTs with affective bias as a covariate showed a main effect of age group ($F(1,76) = 14.40$, $p < .001$, $\eta_p^2 = 0.16$) because SSRTs were significantly longer for older than younger adults, indicating less efficient response inhibition overall in older adults. There was no main effect of emotion ($F(1,77) = 2.73$, $p = .10$, $\eta_p^2 = 0.03$) or interaction of age group

and emotion ($F(1,77) = 0.31$, $p = .58$, $\eta_p^2 = 0.004$). There was no main effect of affective bias on SSRTs ($F(1,76) = 2.85$, $p = .10$, $\eta_p^2 = 0.04$).

Results of analyses on stop-signal delays with affective bias as a covariate indicated no main effect of age group ($F(1,76) = 1.59$, $p = .21$, $\eta_p^2 = 0.02$) or emotion ($F(1,77) = 0.18$, $p = .67$, $\eta_p^2 = 0.002$) or interaction of age group and emotion ($F(1,77) = 0.30$, $p = .59$, $\eta_p^2 = 0.004$). There was no main effect of affective bias on stop-signal delays ($F(1,76) = 0.42$, $p = .52$, $\eta_p^2 < 0.01$).

Results of analyses on go-image accuracy with factors of emotion and age group and covariate of affective bias demonstrated a main effect of age group ($F(1,76) = 10.91$, $p = .001$, $\eta_p^2 = 0.13$) because overall go-image response accuracy was higher for younger than older adults. There was also a main effect of emotion ($F(1,77) = 22.60$, $p < .0001$, $\eta_p^2 = 0.23$) because Go response accuracy was higher for pleasant than unpleasant images. These main effects were qualified by an interaction between age group and emotion ($F(1,77) = 9.04$, $p = .004$, $\eta_p^2 = 0.11$) revealing that younger adults had higher accuracy than older adults when responding to unpleasant go-images, while there were no group differences in response accuracy for pleasant go-images (as reported in main text). There was also a main effect of affective bias on go-image accuracy ($F(1,76) = 4.58$, $p = .04$, $\eta_p^2 = 0.06$).

Considering individual differences in self-reported anxiety did not change interpretation of results

Both state (STAI-S) and trait (STAI-T) anxiety levels (Spielberger and Gorsuch, 1983) were significantly higher in younger than older adults (STAI-S: $t(77) = 13.64$, $p < .001$, $d = 3.07$; STAI-T: $t(69.07) = 3.89$, $p < .001$, $d = 0.87$; descriptive statistics reported in Table S2). In consideration of this distinction and the possibility that it may impact stop-signal task performance, we repeated all task analyses reported in the main text first including state anxiety as a covariate and secondly including trait anxiety as a covariate. To preview effects, the task results did not change when including either measure of self-reported anxiety as a covariate in analyses, as reported below. All significant effects remained significant and null effects remained so after testing the possibility that group differences in anxiety would significantly impact stop-signal task performance.

State Anxiety. The mixed-effects ANOVA of the effects of age and emotion on SSRTs with state anxiety (STAI-S) as a covariate showed a main effect of age group ($F(1,76) = 13.88$, $p < .001$, $\eta_p^2 = 0.15$) because SSRTs were significantly longer for older than younger adults, indicating less efficient response inhibition overall in older adults. There was no main effect of emotion ($F(1,77) = 2.73$, $p = .10$, $\eta_p^2 = 0.03$) or interaction of age group and emotion ($F(1,77) = 0.31$, $p = .58$, $\eta_p^2 = 0.004$). There was no main effect of state anxiety on SSRTs ($F(1,76) = 0.005$, $p = .94$, $\eta_p^2 < 0.001$).

Results of analyses on stop-signal delays with STAI-S as a covariate indicated no main effect of age group ($F(1,76) = 1.64$, $p = .20$, $\eta_p^2 = 0.02$) or emotion ($F(1,77) = 0.18$, $p = .67$, $\eta_p^2 = 0.002$) or interaction of age group and emotion ($F(1,77) = 0.30$, $p = .59$, $\eta_p^2 = 0.003$) on stopping accuracy. There was no main effect of state anxiety on stop-signal delays ($F(1,76) = 2.94$, $p = .09$, $\eta_p^2 = 0.04$).

Results of analyses on go-image accuracy with factors of emotion and age group and covariate of STAI-S demonstrated a main effect of age group ($F(1,76) = 10.42$, $p = .002$, $\eta_p^2 = 0.12$) because overall go-image response accuracy was higher for younger than older adults. There was also a main effect of emotion ($F(1,77) = 22.60$, $p < .0001$, $\eta_p^2 = 0.23$) because Go response accuracy was higher for pleasant than unpleasant images. These main effects were qualified by an interaction between age group and emotion ($F(1,77) = 9.04$, $p = .004$, $\eta_p^2 = 0.11$) revealing that younger adults had higher accuracy than older adults when responding to unpleasant go-images, while there were no group differences in response accuracy for pleasant go-images (as reported in main text). There was no main effect of state anxiety on go-image accuracy ($F(1,76) = 0.99$, $p = .32$, $\eta_p^2 = 0.01$).

Trait Anxiety. The mixed-effects ANOVA of the effects of age and emotion on SSRTs with trait anxiety (STAI-T) as a covariate showed a main effect of age group ($F(1,76) = 13.94$, $p < .001$, $\eta_p^2 = 0.16$) because SSRTs were significantly longer for older than younger adults, indicating less efficient response inhibition overall in older adults. There was no main effect of emotion ($F(1,77) = 2.73$, $p = .10$, $\eta_p^2 = 0.03$) or interaction of age group and emotion ($F(1,77) = 0.31$, $p = .58$, $\eta_p^2 = 0.004$). There was no main effect of trait anxiety on SSRTs ($F(1,76) = 0.36$, $p = .55$, $\eta_p^2 = 0.005$).

Results of analyses on stop-signal delays with STAI-T as a covariate indicated no main effect of age group ($F(1,76) = 1.64$, $p = .20$, $\eta_p^2 = 0.02$) or emotion ($F(1,77) = 0.18$, $p =.67$, $\eta_p^2 = 0.002$) or interaction of age group and emotion ($F(1,77) = 0.30$, $p = .59$, $\eta_p^2 = 0.004$) on stopping accuracy. There was no main effect of trait anxiety on stop-signal delays ($F(1,76) = 2.69$, $p = .10$, $\eta_p^2 = 0.03$).

Results of analyses on go-image accuracy with factors of emotion and age group and covariate of STAI-T demonstrated a main effect of age group ($F(1,76) = 10.32$, $p = .002$, $\eta_p^2 = 0.12$) because overall go-image response accuracy was higher for younger than older adults. There was also a main effect of emotion ($F(1,77) = 22.60$, $p < .0001$, $\eta_p^2 = 0.23$) because Go response accuracy was higher for pleasant than unpleasant images. These main effects were qualified by an interaction between age group and emotion ($F(1,77) = 9.04$, $p = .004$, $\eta_p^2 = 0.11$) revealing that younger adults had higher accuracy than older adults when responding to unpleasant go-images, while there were no group differences in response accuracy for pleasant go-images (as reported in main text). There was no main effect of trait anxiety on go-image accuracy ($F(1,76) = 0.21$, $p = .65$, $\eta_p^2 = 0.003$).

Post-task feedback survey

A 9-point Likert scale of perceived task difficulty administered directly following the stop-signal task showed that on average, participants reported the stop-signal task to be "neither easy nor difficult" (equivalent to rating of 5), with no significant difference between age groups, $t(77) = 0.47$, $p = .64$, $d = 0.11$; older adults $M = 4.88$, younger adults $M = 5.10$. Additionally, self-reports of subjective allocation of their effort to the two competing goals of accurately inhibiting responses on the stop trials and quickly responding on go trials indicated there was no difference in participants' perceived allocation of their efforts between the two goals, $t(76) = 0.38$, $p = .71$, $d = 0.04$, and there was no difference between age groups in their respective efforts to either goal ($t(75) = 0.42$, $p = .68$, $d = 0.10$. Of their 100% total effort, both age groups allocated about 49% of their effort to accurately inhibiting responses on the stop trials and 51% of their effort to quickly responding on go trials. Thus, the perceived stop-signal task difficulty and also relative allocation of effort to both task goals were comparable between younger and older adults.

Supplemental References

Beck, A. T., & Steer, R. A. (1993). *Beck Anxiety Inventory Manual*. San Antonio, TX: The Psychological Corporation Harcourt Brace & Company.

Beck, A. T., Ward, C. H., Mendelson, M., Mock, J., & Erbaugh, J. (1961). An inventory for measuring depression. *Archives of General Psychiatry*, *4*, 561–571. https://doi.org/10.1001/archpsyc.1961.01710120031004

Dan-Glauser, E. S., & Scherer, K. R. (2011). The Geneva affective picture database (GAPED): A new 730-picture database focusing on valence and normative significance. *Behavior Research Methods*, *43*(2), 468–477. https://doi.org/10.3758/s13428-011-0064-1

Delis, D. C., Kaplan, E., & Kramer, J. H. (2001). *Delis-Kaplan executive function system (D-KEFS): Examiner's manual*. San Antonio, TX: The Psychological Corporation.

Folstein, M. F., Folstein, S. E., & McHugh, P. R. (1975). "Mini-mental state": A practical method for grading the cognitive state of patients for the clinician. *Journal of Psychiatric Research*, *12*(3), 189–198. https://doi.org/10.1016/0022-3956(75)90026-6

Kurdi, B., Lozano, S., & Banaji, M. R. (2017). Introducing the Open Affective Standardized Image Set (OASIS). *Behavior Research Methods*, *49*(2), 457–470. https://doi.org/10.3758/s13428-016-0715-3

Pachana, N. A., Byrne, G. J., Siddle, H., Koloski, N., Harley, E., & Arnold, E. (2007). Development and validation of the Geriatric Anxiety Inventory. *International Psychogeriatrics*, *19*(1), 103–114. https://doi.org/10.1017/S1041610206003504

Reitan, R. M. (1958). Validity of the Trail Making Test as an indicator of organic brain damage. *Perceptual and Motor Skills*, *8*(3), 271–276. https://doi.org/10.2466/pms.1958.8.3.271

Spielberger, C. D., Gorsuch, R. L., Vagg, P. R., & Jacobs, G. A. (1983). *Manual for the State-Trait Anxiety Inventory*. Palo Alto, CA: Consulting Psychologist Press.

Verbruggen, F., Aron, A. R., Band, G. P., Beste, C., Bissett, P. G., Brockett, A. T., Brown, J. W., Chamberlain, S. R., Chambers, C. D., Colonius, H., Colzato, L. S., Corneil, B. D., Coxon, J. P., Dupuis, A., Eagle, D. M., Garavan, H., Greenhouse, I., Heathcote, A., Huster, R. J., … Boehler, C. N. (2019). A consensus guide to capturing the ability to inhibit actions and impulsive behaviors in the stop-signal task. *eLife*, *8*, e46323. https://doi.org/10.7554/elife.46323

Yesavage, J. A., Brink, T. L., Rose, T. L., Lum, O., Huang, V., Adey, M., & Leirer, V. O. (1983). Development and validation of a geriatric depression screening scale: A preliminary report. *Journal of Psychiatric Research*, *17*(1), 37–49. https://doi.org/10.1016/0022-3956(82)90033-4
